# Applying the Robson classification to routine facility data to understand the Caesarean section practice in conflict settings of South Kivu, eastern DR Congo

**DOI:** 10.1371/journal.pone.0237450

**Published:** 2020-09-08

**Authors:** Guy Mulinganya, Espoir Bwenge Malembaka, Melissa Lukula Akonkwa, Dieudonné Mpunga Mukendi, Etienne Kajibwami Birindwa, Ghislain Maheshe Balemba, Marleen Temmerman, Albert Mwembo Tambwe, Bart Criel, Ghislain Bisimwa Balaluka

**Affiliations:** 1 Department of Gynecology and Obstetrics, Faculty of Medicine, Université Catholique de Bukavu, Bukavu, Democratic Republic of Congo; 2 Faculty of Medicine and Health Sciences, Ghent University, Ghent, Belgium; 3 Renforcement Institutionnel Pour des Politiques de Santé Basées sur l’Evidence, Democratic Republic of Congo, Lubumbashi, Democratic Republic of Congo; 4 Ecole Régionale de Santé Publique, ERSP, Faculté de Médecine, Univesité Catholique de Bukavu, Bukavu, Democratic Republic of Congo; 5 Institute of Health and Society, IRSS, Ecole de Santé Publique, Université Catholique de Louvain, Brussels, Belgium; 6 School of Public Health, University of Kinshasa, Kinshasa, Democratic Republic of Congo; 7 Department of Radiology, Faculty of Medicine, Université Catholique de Bukavu, Bukavu, Democratic Republic of Congo; 8 Centre of Excellence in Women and Child Health, School of Medicine, Aga Khan University, Nairobi, Kenya; 9 Ecole de Santé Publique, Université de Lubumbashi, Lubumbashi, Democratic Republic of Congo; 10 Institute of Tropical Medicine, Antwerp, Belgium; 11 Université du Cinquantenaire de Lwiro, Lwiro, Democratic Republic of Congo; Lausanne University Hospital: Centre Hospitalier Universitaire Vaudois (CH), SWITZERLAND

## Abstract

**Introduction:**

Sub-Saharan Africa has low Caesarean (CS) levels, despite a global increase in CS use. In conflict settings, the pattern of CS use is unclear because of scanty data. We aimed to examine the opportunity of using routine facility data to describe the CS use in conflict settings.

**Methods:**

We conducted a facility-based cross-sectional study in 8 health zones (HZ) of South Kivu province in eastern DR Congo. We reviewed patient hospital records, maternity registers and operative protocol books, from January to December 2018. Data on direct conflict fatalities were obtained from the Uppsala Conflict Data Program. Based on conflict intensity and chronicity (expressed as a 6-year cumulative conflict death rate), HZ were classified as unstable (higher conflict death rate), intermediate and stable (lower conflict death rate). To describe the Caesarean section practice, we used the Robson classification system. Based on parity, history of previous CS, onset of labour, foetal lie and presentation, number of neonates and gestational age, the Robson classification categorises deliveries into 10 mutually exclusive groups. We performed a descriptive analysis of the relative contribution of each Robson group to the overall CS rate in the conflict stratum.

**Results:**

Among the 29,600 deliveries reported by health facilities, 5,520 (18.6%) were by CS; 5,325 (96.5%) records were reviewed, of which 2,883 (54.1%) could be classified. The overall estimated population CS rate was 6.9%. The proportion of health facility deliveries that occurred in secondary hospitals was much smaller in unstable health zones (22.4%) than in intermediate (40.25) or stable health zones (43.0%). Robson groups 5 (previous CS, single cephalic, ≥ 37 weeks), 1 (nulliparous, single cephalic, ≥ 37 weeks, spontaneous labour) and 3 (multiparous, no previous CS, single cephalic, ≥ 37 weeks, spontaneous labour) were the leading contributors to the overall CS rate; and represented 75% of all CS deliveries. In unstable zones, previous CS (27.1%) and abnormal position of the fetus (breech, transverse lie, 3.3%) were much less frequent than in unstable and intermediate (44.3% and 6.0% respectively) and stable (46.7%and 6.2% respectively). Premature delivery and multiple pregnancy were more prominent Robson groups in unstable zones.

**Conclusion:**

In South Kivu province, conflict exposure is linked with an uneven estimated CS rate at HZ level with at high-risks women in conflict affected settings likely to have lower access to CS compared to low-risk mothers in stable health zones.

## Background

It is commonly heralded that a caesarean section (CS) rate between 10–15% is associated with public health benefits for mothers and new-borns [1, 2]. Globally, there is a growing recognition of CS as an emergency and essential surgical care, thus a key component of universal health coverage. The World Health Organisation (WHO) statement on CS rate [3] and the Lancet commission on global surgery [4] have all renewed the pressing call for investment in obstetric surgery and provision of CS to all women in need. However, unlike in many middle and high income settings where the CS rate has excessively increased beyond what is believed adequate, sub-Saharan Africa remains a region with the lowest and suboptimal CS levels [5, 6]. The 2018 Lancet series on CS reported a 14-fold higher use of CS in the Dominican Republic (58%) compared to the average of 4.1% observed in west and central African countries of which the Democratic Republic of Congo (DRC) is part [7]. In addition to the low rate, low-income settings bear a disproportionately high burden of perinatal complications of CS [8]. A recent study reported a 50-fold higher maternal mortality associated with delivery by CS in Africa compared to high income countries [9].

In conflict and post-conflict regions of Africa, populations are impoverished and illiterate [10], fertility rates high [11, 12] and there is often an inextricable coexistence of (lack of clear) governments agendas [13], faith-based actors and humanitarian donors’ actions with sometimes diverging priorities [14], as it is the case in eastern DRC [15]. The conflict in the Kivu region (DRC) has lasted for decades and has had destructive direct and indirect health effects [16–18]. Though the war in DRC officially ended in 2002 [19], there were about 140 active armed groups still operating in the Kivu in 2019 [20]. In such a context of protracted conflict, the blurred landscape of the CS use and its outcomes is compelling and requires a special attention.

The 2018 DRC Multiple Indicator and Cluster Survey (MICS) estimated the CS rate at 4.7% nationwide; the Kivu region had noticeably higher CS rates (12.3% in North Kivu and 10.8% in South Kivu) [21]. Higher coverage rates were also reported for other interventions indicators in the reproductive, maternal, new-born and child health (RMNCH) continuum [22]. But whether and to which extent the protracted Kivu conflict has influenced the use of CS across and within health zones of the Kivu provinces is yet to be fully assessed.

A recent nationwide survey suggested that only 2.9% of health facilities provided comprehensive emergency obstetric care (CEmOC) in DRC, moreover, of low quality [22]. In this study, we aimed to describe the patterns of CS rate vis-à-vis exposure to armed conflict, by applying the Robson classification system [23] to routine data from secondary health facilities in South Kivu. Our ultimate goal is to inform strategies towards a universal and optimal access to CS for the women most in need in eastern DRC and other similar fragile contexts.

## Methods

### Study design, settings and population

We conducted a facility-based cross-sectional study in 8 of the 34 health zones (HZ) of South Kivu, covering 27.6% of the estimated 6,937,726 inhabitants of this province in 2018, based on population projections from the South Kivu provincial health division. The selection of the study sites was based on a priori geographical (rural versus urban, accessible versus hard-to-reach health zones), security and health systems criteria (reported levels of CS and presence of humanitarian actors). Bunyakiri, Fizi, Idjwi, Kadutu, Kalehe, Mulungu, Mwana and Walungu health zones were selected. Fizi HZ has long remained a hotspot of conflict and insecurity [24], and is the focus of substantive humanitarian action [25]. Mulungu HZ is also armed groups-prone and one of the hardest-to-reach HZ of South Kivu, but has a relatively weak presence of humanitarian actors as opposed to Fizi. Idwi HZ is the largest island of the Kivu Lake and has been relatively spared from direct effects of the decades-long eastern DRC conflict. Kadutu is a semi-urban health zone located in Bukavu, the capital city of South Kivu. Bunyakiri, Kalehe, Mwana and Walungu are rural HZ recovering from conflict with varying presence of internally displaced persons and humanitarian actors subsidizing C-section services [25].

#### Definition of exposure to armed conflict

The Uppsala Conflict Data Program Georeferenced Events Dataset (UCDP GED) was the main source of conflict exposure data. Georeferenced conflict events and deaths data were merged with the shape files of the 516 DRC health zones obtained from the United Nations Office for Coordination of Humanitarian Affairs (UNOCHA) with the use the of QGIS 3.4 software. For each of the eight health zones included in this study, the number of total deaths directly attributable to conflict was used to calculate a cumulative death rate. In fact, the number of total fatalities recorded in 2018 and the five previous years was divided by the average annual population estimate to capture the intensity and chronicity of conflict. Fizi and Mulungu health zones had a cumulative conflict death rate of 51.7 and 21.0 per 100,000 inhabitants and were classified as unstable or insecure. Bunyakiri and Kalehe had a death rate of 2.4 and 8.3 per 100,000 and were considered intermediate. All the other HZ had a death rate of 0.4 per 100,000 or lower and were treated as stable or least insecure.

Within each health zones, secondary health facilities that officially offered C-section services throughout 2018 were targeted. In Kadutu health zone, data collectors were denied access to hospital records in one health facility affiliated with a public university. Two more health facilities were not accessible because of active conflict in Fizi and completely impassable forested roads in Mulungu.

The study population under this study is comprised of women who delivered by Caesarean section in 2018, based on health facility records from health facilities in these 8 health zones.

*Data collection and the Robson classification*. A structured questionnaire was used by 8 data collectors who were all medical doctors trained on the use of the Robson classification system. The latter was first proposed in 2001 as a method for assessing the CS rate among women with various obstetrical characteristics [26]. It has proved to be an objective and standardised tool for monitoring, auditing, and comparison of CS rate trends across institutions, regions and over time [27–31]. Records review included patient hospital records, maternity and operating rooms registers and protocol books, from January to December 2018. Data were sought for sociodemographic characteristics, obstetrical history of mothers who delivered by CS and new-borns characteristics. Data collectors were assigned to classify women with sufficient information into the 10 Robson groups. The classification work was then cross-checked, corrected with Microsoft excel and validated by a supervisor who was also a medical doctor. The WHO has declared the Robson classification a standardized tool for analysing trends in CS rates within and between health facilities globally [3]. Based on parity, history of previous CS, onset of labour, foetal lie and presentation, number of neonates and gestational age, the Robson classification categorises deliveries into 10 mutually exclusive groups.

#### Analysis of CS practise using the Robson classification

The main study outcome was the relative contribution of each Robson group to the overall CS delivery rate, expressed as the number of women with a CS delivery in a group divided by the total number of CS that could be correctly classified. The secondary outcome was the estimated population CS rate. Pearson’s chi squared test was used to compare the sociodemographic and healthcare characteristics of mothers and new-borns. We used the 10-group Robson classification to describe the Caesarean section practice [26]. For each Robson class, we report the relative contribution on the overall CS rate in terms of the number of CS in the group over the total number of CS in the conflict category.

#### Estimating the population CS rate

We applied the crude birth rate (CBR) reported in the latest 2018 DRC MICS (46.0 and 37.5 live births for 1000 population in rural and urban areas respectively) to the 2018 population projections from the South Kivu Ministry of Health to estimate the population CS rate. The 2018 DRC MICS also estimated the health facility delivery proportion at 93.5% in South Kivu [21]. The numerators for CS rates estimates were CS counts from studied secondary-level health facilities and the online District Health Information System (DHIS2) at health zone level. We report CS rate estimates using both CS count data collected from secondary level health facilities and HZ data available in the DHIS2 to account for under or over reporting. The alpha error margin was set to p<0.05. Analyses were done with Stata 15.

### Ethical consideration

The protocol of this non-human subject study was approved by the ethics committee of the Université Catholique de Bukavu. Permission to access facility records was obtained from the South Kivu Ministry of Health (MoH) and health facilities officials.

## Results

Thirty secondary health facilities were successfully investigated, of which 11 were fully government-owned, 14 were run by faith organisations and 5 were private. From these facilities, 5520 CS (18.6%) out of 29,600 deliveries were reported, of which 5325 records were available, corresponding to 96.4% of the CS deliveries and 18.0% of all the deliveries recorded.

### General characteristics mothers and new-borns

Table 1 displays the socio-demographic and healthcare characteristics of mothers and new-borns. Fourteen percent of the study population were adolescents and this proportion tended to increase with the level of insecurity (p<0.001). While 16.9% of the study population had a history of at least one abortion, 27.7% were nulliparous and 22.1% had a history of at least one death in the offspring; the proportion of mothers with at least two deaths in the offspring in unstable HZ was over double (12%) than that in stable HZ (5.9%). A history of previous CS was common in 55.8% percent of the study population with an inverse downward trend along the conflict gradient. In unstable HZ, the level of preterm births (13.7%) was 2.4-fold and 4.5-fold higher than that in intermediate and stable health zones respectively (p<0.001). Likewise, low birth weight in unstable HZ (15.3%) was 1.9 and 1.8 times more common than in intermediate and stable HZ respectively (p<0.001). Only 20.8% of women reported having a health coverage among those with available data (n = 1929), and this number was remarkably higher (p<0.001) in unstable HZ (72.2%) than in intermediate (9.3%) and stable ones (5.8%).

**Table 1:** Socio-demographic and healthcare characteristics of mothers and new-borns.

Variable	Total	Stable	Intermediate	Unstable	P value
Maternal age (year), n = 4823					<0.001
<20	676 (14.0)	284 (10.1)	246 (18.4)	146 (21.8)	
20–34	3520 (73.0)	2151 (76.4)	918 (68.8)	451 (67.2)	
35+	627 (13.0)	382 (13.5)	171 (12.8)	74 (11.0)	
Abortion, n = 3420					0.002
0	2842 (83.1)	1513 (83.4)	840 (84.3)	489 (80.3)	
1	421 (12.3)	204 (11.3)	116 (11.6)	101 (16.6)	
2+	157 (4.6)	97 (5.4)	41 (4.1)	19 (3.1)	
Parity, n = 3673					<0.001
0	1018 (27.7)	585 (28.9)	235 (23.4)	198 (30.9)	
1	599 (16.3)	348 (17.2)	132 (13.1)	119 (18.6)	
2+	2055 (56.0)	1092 (53.9)	639 (63.5)	324 (50.6)	
Previous C-section, n = 4,065					<0.001
No	1798 (44.2)	887 (40.3)	646 (47.6)	265 (52.7)	
Yes	2267 (55.8)	1317 (59.8)	712 (52.4)	238 (47.3)	
Gestational age (weeks), n = 3273					<0.001
<37	168 (5.1)	57 (3.0)	56 (5.7)	55 (13.7)	
37–38	1015 (31.0)	486 (25.9)	383 (38.7)	146 (36.2)	
39–41	1879 (57.4)	1229 (65.4)	467 (47.1)	183 (45.4)	
42+	211 (6.5)	107 (5.7)	85 (8.6)	19 (4.7)	
Birth weight, n = 4748					<0.001
<2500	446 (9.4)	240 (8.7)	108 (8.1)	98 (15.3)	
2500–2999	1151 (24.2)	637 (23.0)	279 (21.0)	235 (36.0)	
3000–3999	2755 (58.0)	1647 (59.5)	814 (61.3)	294 (45.1)	
4000+	396 (8.3)	244 (8.8)	127 (9.6)	25 (3.8)	
Death history in the offspring, n = 3404					<0.001
Zero	2651 (77.9)	1510 (84.1)	726 (73.0)	415 (67.7)	
One	453 (13.3)	181 (10.1)	150 (15.1)	122 (19.9)	
Two or more	300 (8.8)	105 (5.9)	119 (12.0)	76 (12.4)	
Health coverage, n = 1929					<0.001
No	1528 (79.2)	975 (94.2)	439 (90.7)	114 (27.8)	
Health coverage	401 (20.8)	60 (5.8)	45 (9.3)	296 (72.2)	

Data are n (%). The sample size varies between variables depending on the completeness of data sources.

### Description of the CS practice using the Robson classification

We assumed that the all health zones had the same institutional delivery rate to obtain the estimated number of deliveries in the population. Based on this denominator, the distribution of C-section based on the Robson classification is presented by conflict location in Table 2. Of the 5235 CS recorded and reviewed, 2883 (54.1%) could be correctly classified. Overall, Robson groups 5 (44.1%), 1(19.7%) and 3 (14.7%) were the leading single contributors to the overall CS rate and altogether represented 75% of all CS. The contribution of group 1 appeared identical across conflict locations whereas group 3 seemed to contribute more to overall CS rate in unstable (19.8%) and intermediate (19.4%) than in stable HZ (11.4%). Remarkably, 46.7% and 44.3% of CS in stable and intermediate HZ respectively concerned the Robson group 4 (Multiparous with previous Caesarean section, singleton, cephalic, ≥37 weeks’ gestation) while this group contributed only 27.1% of the CS rate in unstable HZ. Robson group 10 (preterm births) accounted for up to 13.2% of the CS rate in unstable HZ against only 2% in stable HZ and 5% in intermediate HZ.

**Table 2:** Distribution of C-section by conflict location using the Robson classification.

Robson groups	Description of Robson groups	Stable HZ	Intermediate HZ	Unstable HZ	Total
**1**	Nulliparous, singleton, cephalic, ≥37 weeks’ gestation, in spontaneous labour	341 (19.9)	171 (19.1)	56 (20.5)	568 (19.7)
**2**	Nulliparous, singleton, cephalic, ≥37 weeks’ gestation, induced labour or Caesarean section before labour	46 (2.7)	6 (0.7)	7 (2.6)	59 (2.1)
**3**	Multiparous (excluding previous CS), singleton, cephalic, ≥37 weeks’ gestation, in spontaneous labour	196 (11.4)	173 (19.4)	54 (19.8)	423 (14.7)
**4**	Multiparous without a previous CS, singleton, cephalic, ≥37 weeks, induced labour or Caesarean section before labour	44(2.6)	4.0 (0.5)	6.0 (2.2)	54 (1.9)
**5**	Previous CS, singleton, cephalic, ≥37 weeks	801 (46.7)	396 (44.3)	74 (27.1)	1271 (44.1)
**6**	All nulliparous with a single breech	27 (1.6)	20 (2.2)	2 (0.7)	49 (1.7)
**7**	All multiparous with a singleton breech (including previous CS)	23 (1.3)	27 (3.0)	3 (1.1)	53 (1.8)
**8**	All multiple pregnancies (including previous CS)	146 (8.5)	43 (4.8)	31 (11.4)	220 (7.6)
**9**	All singleton, transverse or oblique lie (including CS section)	57 (3.3)	7 (0.8)	4 (1.5)	68 (2.4)
**10**	All singleton, cephalic, <37 weeks (including previous CS)	35 (2.0)	47 (5.3)	36 (13.2)	118 (4.1)

The unstable health zones have much lower proportion of institutional deliveries taking place in secondary health facilities with CS capacity: 22.4% of all facility deliveries compared to 40.2% and 43.0% in intermediate and stable health zones respectively.

### Description and comparison of CS levels by conflict location

We assumed that the all health zones had the same institutional delivery rate to obtain the estimated number of deliveries in the population. Based on this denominator, the estimated CS rate at population level was 6.9% in the 8 health zones of study. Unstable health zones had markedly lower CS levels (4.1%) than intermediate (8.6%) and stable zones (7.6%). In fact, the estimated population CS rate in unstable zones appeared almost half that in intermediate zones and stable health zones. The health facility CS rate ranged from 12.4% in Idjwi (a stable health zone) to 32.9% in Mulungu (unstable health zone) (Table 3). However, Idjwi (island) and Mulungu health zone (the remotest), despite having a different security history, appeared to have the lowest population CS rates. The unstable health zones have much lower proportion of institutional deliveries taking place in secondary health facilities with CS capacity: 22.4% of all facility deliveries compared to 40.2% and 43.0% in intermediate and stable health zones respectively.

**Table 3:** Estimated health facility delivery and population CS rate estimates by conflict location.

Health zones	2018 Population	Estimated births in HZ	Estimated health facility births	SHF deliveries	SHF CS	Population CS	
						With DHIS2 numerator	With SHF numerator
Stable	1060620	45645	42679	18347	3272(17.8)*	3057 (7.1)	7.6%
Idjwi	281983	12971	12128	3411	424 (12.4)	380 (3.2)	3.5%
Kadutu	369712	13864	12963	7382	1245 (16.9)*	1136 (8.2)	9.0%
Mwana	139332	6409	5993	3323	862 (25.9)	731 (12.3)	14.6%
Walungu	269593	12401	11595	4231	741 (17.5)	810 (7.1)	6.5%
Intermediate	392878	18072	16898	6797	1438 (21.2)	1386 (8.3)	8.6%
Bunyakiri	212083	9756	9122	2913	772 (26.5)	898 (10.0)	8.6%
Kalehe	180795	8317	7776	3884	666 (17.1)	488 (6.4)	8.7%
Unstable	462906	21294	19910	4456	810 (18.2)	1044 (5.3)	4.1%
Fizi	310755	14295	13366	3681	555 (15.1)*	789 (6.0)	4.2%
Mulungu	152151	6999	6544	775	255 (32.9)*	255 (3.9)	3.9%
Total	1916404	85012	79486	29600	5520 (18.6)*	5487 (6.9)	6.9%

SHF: secondary-level health facility. Data are n and n (%), unless otherwise specified. *: The number of CS deliveries performed in the 3 health facilities that could not be accessed were obtained from the DHIS2 to allow more accurate estimates (104 in Kadutu, 8 in Fizi and 85 in Mulungu). Data are n and n (%), unless otherwise specified. Secondary-level health facilities (SHF) are those that have a permanent medical doctor, and can officially offer hospitalization and CS services. The crude birth rate (CBR) reported in the latest (2018) DRC MICS (46.0 and 37.5 live births for 1000 population in rural and urban areas respectively) was applied to the 2018 population projections from the South Kivu Ministry of Health. The 2018 DRC MICS also estimated the health facility delivery proportion at 93.5% in South Kivu. The numerators for CS rates estimates were CS counts from studied SHF and the online District Health Information System (DHIS2) at health zone level.

### Missing data pattern

The pattern of missing data was uneven across variables (Table 4). Of the core Robson system variables, gestational age was the most concerned by missing data (38.5%) while the number of foetuses was the most reported parameter with 11.6% of missing data.

**Table 4:** Distribution of missing data by crisis location.

Variable	Stable	Intermediate	Unstable	Total	P value
Robson	1452 (45.3)	544 (37.8)	446 (62.0)	2442 (45.9)	<0.001
Maternal age	351 (11.1)	103 (7.16)	48 (6.7)	502(9.4)	<0.001
Abortion	1354 (42.7)	422 (30.7)	112 (15.6)	1908 (35.8)	<0.001
Parity	1142 (36.1)	432 (30.0)	78 (10.9)	1652 (31.0)	<0.001
Previous CS	878 (27.7)	69 (4.8)	210 (29.2)	1157 (21.7)	<0.001
Gestational age	1289 (40.7)	447 (31.1)	316 (43.9)	2052 (38.5)	<0.001
Number of foetuses	493 (15.6)	45 (3.13)	81 (11.3)	619 (11.6)	<0.001
Presentation	1124 (35.5)	470 (32.7)	151 (21.0)	1745 (32.7)	<0.001
Onset of labour	1210 (38.2)	391 (27.2)	268 (37.3)	1869 (35.1)	<0.001
Birth weight	400 (12.6)	110 (7.7)	67 (9.3)	557 (10.8)	<0.001
Death history in the offspring	1372 (43.3)	443 (30.8)	106 (14.7)	1921 (36.1)	<0.001
Health coverage	2131 (67.3)	953 (66.3)	309 (43.0)	3393 (62.7)	<0.001

Data are n (%) of missing data.

## Discussion

In this facility-based cross-sectional study, overall, the estimated population CS rate was 6.9% and appeared higher in intermediate health zones compared to stable and unstable locations. The proportions of adolescent mothers, preterm births, new-borns with low birth weight and mothers with history of deaths in the offspring appeared significantly higher in unstable locations. The Robson group 5 was relatively the largest single contributor to the CS rate in all the three conflict contexts.

A number of patterns in our data indicate a differential use of CS based on whether a woman lives in conflict-affected HZ or not. The level of CS use observed in this study appeared lower than the South Kivu average estimated at 10.8%, but higher than the national average (4.1%) [32]. This finding can be explained by lower availability and limited access to CS and other CEmOC services in conflict-affected or rural settings representing the majority of the health zones included in this study. Higher CS use level in conflict and post-conflict health zones than the national average (5.1%) indicates that other health systems factors than conflict play a critical role [21, 33]. The lower CS use observed in unstable areas could reflect the lack of healthcare facilities and the poor access to health services. The higher CS rate in intermediate zones may be partly explained by the strong presence of humanitarian actors in these locations on the one hand [34], and a lower intensity of conflict compared to unstable locations on the other. The presence of humanitarian action may result in better funding of the local health system, and better retention rate of qualified health workers, reporting and quality data keeping, and maternal and child health coverage indicators respectively. Lastly, the lower CS rate in unstable HZ may allow some women to avoid the high morbidity and mortality risk associated with CS in low-income countries [9].

Of note was also the larger size of Robson group 10 (preterm deliveries) in unstable HZ, in line with worse mothers and new-borns health characteristics observed in conflict locations, notably adolescent expecting mothers, low birth weight and preterm babies and deaths in mothers’ offspring. This trend would have even been more marked if data incompleteness on gestational age was not higher in unstable locations compared to stable ones. One of the rare studies focusing on pregnancy outcomes in eastern DRC pointed to a two-fold increase in the rate of preterm births in a hospital following the eruption of the 1997 war in the now Ituri province [35], consistent with the literature from other conflict-plagued contexts [36]. Inaccurate estimation of gestational age, mostly based on clinical evaluations and last menstrual periods is possible [37], though the latter was suggested to be a reliable alternative for the assessment of gestational age in low-income settings where early pregnancy ultrasound is not readily accessible [38].

The size of Robson group 8 appeared larger than the expected number of multiple pregnancies in the general population [39]. This finding is in agreement with the high level of preterm births observed in health facilities surveyed, given the high likelihood of preterm deliveries in women with multiple pregnancies. This result may also be indicative of doctors’ conservative propensity vis-à-vis CS in mothers with twin pregnancies in resource-constrained settings where maternal and child mortality and morbidity associated to multiple pregnancy delivery is high [40]. This result could also indicate a likely high twinning rate in South Kivu; further studies are needed to support this hypothesis.

Noticeably, in unstable zones the size of group 5 (iterative Caesarean section) appeared remarkably lower compared to that in stable and intermediate HZ. This trend is suggestive of an inverse care law pattern [41] whereby at-high-risk mothers living in insecure locations have less access to CS in case of need. Problematic geographical and financial accessibility, insecurity, lack of human resources–even with doctors being killed or fleeing conflict locations—may contribute to this state of affairs.

### Study limitations and strengths

Using data from secondary health facilities, particularly in contexts where access to CEmOC is problematic, precludes the generalisability of this study to all the women in need of a CS in the HZ studied. The insufficiency of data did not allow us gain a deeper insight into factors driving the CS demand and accessibility. Some health centres and health posts (officially run by nurses) in DRC, mainly in rural and hard-to-reach areas, illicitly provide CS and blood transfusion services despite obvious lack in capacity to do so [33]. CS performed in these primary health care facilities are not accounted for in our analysis. We hypothesize that mothers delivering at health post or health centre level face major problems in accessing higher-level facilities offering CS and other CEmOC services in better circumstances.

Another threat to the generalisability of our results is the inclusion of only 8 health zones out of the 34 HZ that compose the South Kivu province. Some health facilities were not accessible because of insecurity and physical inaccessibility. These geopolitically enclosed areas are likely to have a different CS rate pattern and worse maternal and child health outcomes.

Of concern was also the sub-optimal records keeping in many health facilities, hindering the application of the Robson classification to a larger number of mothers. Neither could we provide insights into the other dimensions of the Robson classification, that is, the CS rate within each Robson group or the relative size of each group. The limited data availability also impeded more detailed analysis on the socio-demographic and health systems drivers of different CS patterns observed between and within conflict strata. For instance, the huge CS rate difference between Idjwi and Mwana, two rural and stable health zones, suggests that other factors than conflict play a critical role. Likewise, analysis of maternal and child outcomes of CS was unfeasible. Future studies based on prospective data at a larger scale would be needed to examine how factors such as educational attainment and socio-economic position interrelate with conflict in the expression of the CS patterns. Neither could we assess the absolute contribution of each Robson group given the fact that we did not have data to classify the profile of all women delivering within the health facilities studied.

Another limitation of this study is that our conflict variable does not take into account population displacements because of lack of data on internally displaced persons disaggregated by health zone. The cumulative death rate measure we used does not fully capture indirect effects of conflict and other forms of insecurity and violence exerted through abductions of children and women, sexual violence, illegal taxation, kidnappings, etc [24].

In spite of these limitations, our study provides a unique insight into the CS practice in conflict-affected health zones in eastern DRC. As far as we are aware, for the first time, we document the potential for applying the Robson classification system to routine service provision data from health facilities in the Kivu to help understand the drivers and singularities of CS use among the most vulnerable of the vulnerable women, those affected by lingering armed conflicts.

## Conclusion

In South Kivu province, conflict exposure seems to contribute to an uneven CS rate at HZ level with at high-risks women in conflict affected settings likely to have lower access to CS compared to low-risk mothers in stable health zones. The road towards an optimal use of CS both in stable and insecure settings will certainly need to pass by a political will to create peace and improve access to quality CS in the regions ravaged by conflict. Strategies to improve routine service provision data collection are needed to enhance their usefulness in providing a solid basis for monitoring and appraisal of progress towards the optimal use of CS in DR Congo and other low-income countries. Additional research based on prospective data will also be necessary to generate robust and more generalisable evidence for programming and actions towards ensuring that all women, including in conflict settings, have access to quality CS.
